# Machiavellianism and psychopathy affect sleep quality in people with affective disorders and mentally healthy individuals

**DOI:** 10.3389/fpsyg.2023.1248931

**Published:** 2023-09-14

**Authors:** Elena M. D. Schönthaler, Nina Dalkner, Dirk von Lewinski, Eva Z. Reininghaus, Andreas Baranyi

**Affiliations:** ^1^Clinical Department of Psychiatry and Psychotherapeutic Medicine, Medical University of Graz, Graz, Austria; ^2^Clinical Department of Cardiology, Medical University of Graz, Graz, Austria

**Keywords:** dark triad, narcissism, depression, bipolar disorder, health behavior

## Abstract

**Introduction:**

Deteriorated sleep quality is a predisposing factor and symptom of affective disorders (AD). It is important to investigate factors driving the relationship between sleep and AD, such as personality traits. Previous research has shown that personality traits such as the Dark Triad personality traits (DT) narcissism, Machiavellianism, and psychopathy are associated with sleep problems and AD. The current study examined the moderating influence of the DT in the relationship between AD [versus healthy controls (HC)] and sleep quality.

**Methods:**

Data of 657 individuals (267 HC, 390 AD; 483 female, 166 male, eight diverse; *M*_age_ = 34.87, *SD*_age_ = 13.86) were collected in an online survey, which administered the Pittsburgh Sleep Quality Index and the Short Dark Triad questionnaire.

**Results:**

Moderation analyses controlling for age and gender revealed that Machiavellianism (*b* = −0.76, *p* < 0.05, *R*^2^ = 0.35) and psychopathy (*b* = −1.15, *p* < 0.05, *R*^2^ = 0.35), but not narcissism (*b* = −0.20, *p* = 0.620, *R*^2^ = 0.35), had a negative effect on sleep quality. Specifically, this effect is more pronounced in the HC group, but sleep quality is generally worse in AD.

**Conclusion:**

Our findings indicate that Machiavellianism and psychopathy should be considered in the prevention and treatment of AD-associated sleep problems. Particularly, monitoring these traits could help to implement timely measures for the prevention of sleep problems, such as psychoeducation and sleep hygiene. The results highlight the role of personality in the aetiopathogenesis of AD and require further differentiation to examine the underlying pathways between the DT, sleep, and AD.

## 1. Introduction

Affective disorders (AD) subsume psychiatric diseases, which share the symptom of mood dysregulations. For example, depression encompasses depressed mood and/or loss of pleasure or interest. Bipolar disorder includes both depressive and manic episodes, the latter subsuming euphoria and/or heightened irritability. Both share the symptom of disturbed sleep, e.g., an increased or decreased need for sleep, insomnia, or difficulties falling or remaining asleep (World Health Organization, 2022). Hence, the association between AD and sleep has been frequently investigated, revealing that disturbed sleep serves as a symptom and risk factor in AD (Fang et al., 2019). Specifically, impaired sleep continuity and decreased sleep efficiency was found in depression (e.g., Nutt et al., 2008). In bipolar disorder, which is characterized by a decreased need for sleep in manic episodes, sleep onset latency, and variability of sleep–wake patterns are impaired (e.g., Ng et al., 2015). Disturbed sleep is associated with maladaptive health behaviors, which heighten the risk of somatic and psychiatric comorbidities (Gold and Sylvia, 2016). Particularly, sleep disturbances in AD are connected to increased symptom severity, illness duration (e.g., Gruber et al., 2011), and higher suicidality (e.g., Palagini et al., 2019). Contrary, affecting sleep rhythms has been found to positively impact the trajectory of AD, which is reflected by sleep deprivation and light therapy treatments, and sleep hygiene (Walsh et al., 2014; Dallaspezia and Benedetti, 2015; Rahimi et al., 2016). Due to the role of sleep in the etiology and preservation of AD, it is necessary to understand the factors driving the relationship between sleep and AD. Previous research found that sleep disturbances and AD are associated with inflammatory processes, genetic alterations, circadian rhythm interruptions, and dys-balances in biochemical pathways (e.g., Fang et al., 2019). Moreover, social factors (i.e., lifestyle; Lopresti et al., 2013) impact sleep quality, for instance, sleep quality was found to be affected by low-intensity exercise, which also leads to better cognitive functioning (Taheri and Irandoust, 2019), overall performance and well-being (Khcharem et al., 2022), and more regulated appetite (Irandoust and Taheri, 2018). These aspects are usually impaired in individuals with AD (Dalkner et al., 2021; Platzer et al., 2021). Further, psychological variables like personality traits affect both AD and sleep quality (Huang et al., 2016).

The Dark Triad traits (DT) subsume the personality traits narcissism, Machiavellianism, and psychopathy. Narcissism is characterized by grandiosity, entitlement, and superiority, while Machiavellianism encompasses cold and manipulative behavior. Psychopathy includes impulsive behavior along with a lack of empathy and remorse. Despite their unique intra-and interpersonal correlates, all traits share the common core of a socially malevolent character (Paulhus and Williams, 2002). A growing body of literature has linked the DT to dysfunctional interactional and health-related behavior, among others, disturbed sleep habits (e.g., Jonason et al., 2015a; Malesza and Kaczmarek, 2019; Dębska et al., 2021). It was observed that the DT are related to circadian rhythm disturbances and an eveningness disposition (i.e., being most active in the evening; Jonason et al., 2013; Rahafar et al., 2022). Specifically, studies examining the relationship between narcissism and sleep either indicate no association (e.g., Sabouri et al., 2016), or a positive association between narcissism and sleep disturbances (e.g., Ellison et al., 2013). Relatedly, individuals high Machiavellianism and psychopathy reported poorer sleep quality, more sleep disturbances, and insomnia symptoms (Sabouri et al., 2016; Akram et al., 2018; Yang et al., 2019).

Next to a higher risk for sleep disturbances, the DT were associated with AD (Jonason et al., 2015b; Shih et al., 2021). This finding was reattracted to deteriorated emotion regulation strategies (Shen, 2022), but recent research indicated other possible relations between the DT and AD symptoms: Narcissism was found to be a protective factor acting against depressive symptoms, due to higher resilience against distress (e.g., Shih et al., 2021). For Machiavellianism, positive associations with depressive symptoms were found (Bianchi and Mirkovic, 2020). Psychopathy was shown to be positively correlated with depression, which was explained by the fact that externalization of negative moods can result in psychopathic behavior (Gómez-Leal et al., 2019). Contrarily, other studies observed that some psychopathic traits can serve as a protective factor in the development of depression, because they are related to lower stress-levels (e.g., Dalkner et al., 2018).

Considering the investigated links between the DT, sleep, and AD, it seems plausible that these constructs are interrelated. Augmenting knowledge on these relationships may benefit more personalized treatment of sleep disorders, which is fundamental in treating AD. Moreover, it is necessary to conduct research on personality traits, sleep, and AD, since findings within this context could help to identify personality-related aspects, which affect sleep quality. This, in turn, provides another possibility for monitoring and preventing sleep deterioration, which would otherwise worsen or elicit mood-related symptoms. The current study thus aims to extend knowledge on the interrelation between AD (as opposed to healthy controls, HC), the DT, and sleep quality, which is encompasses sleep initiation, sleep maintenance, and sleep quantity (Kline, 2013). Previous findings indicate that Machiavellianism and psychopathy have a negative effect on sleep quality, while there are mixed results for narcissism. However, due to the common core of the Dark Triad (e.g., Book et al., 2015; Bader et al., 2022), we assumed that narcissism would also deteriorate sleep quality in our study. Overall, we hypothesized that the individual manifestations of (a) narcissism, (b) Machiavellianism, and (c) psychopathy significantly negatively moderate the relationship between group (i.e., AD and HC) and sleep quality.

## 2. Materials and methods

### 2.1. Sample and procedure

Participants were recruited at the University Clinic for Psychiatry and Psychotherapeutic Medicine, via a recruiting company (probando.io), and social media. *Post hoc* power calculations using G*Power (version 3.1; Faul et al., 2007) indicated a minimum sample size of 485 participants to detect a small effect (*f*^2^ = 0.02) at an α of 5% and a power of 80%, when considering two predictors. In total, we collected 992 data sets, however, 114 of them were incomplete and excluded from further analyses, yielding data of 878 individuals (HC = 478, AD = 400). Data were included into analyses if participants were between the ages of 18–90 and had a diagnosed AD (in the AD group), or for HC, did not present a diagnosed psychiatric disorder, intake of psychopharmacological drugs, first-degree relatives with diagnosed psychiatric disorders, or undiagnosed life-time depressive symptoms. The presence of a diagnosed AD was assessed with two questions (“*Do you have the diagnosis of an affective disorder?*” and “*Was this diagnosis made by a psychiatrist/psychologist/psychotherapist?*”). Six individuals from the HC group stated a diagnosed psychiatric disorder and psychopharmacological drug intake, and further 31 stated to have a first-degree relative with a diagnosed psychiatric disorder and were thus excluded. To exclude HC participants with life-time depressive symptoms, we used the dichotomous questions of the structured clinical interview for DSM-IV disorders. Both items screen for the main symptoms of depressed mood and loss of joy/interest in the past for a period of at least 2 weeks every day (Wittchen et al., 1997), leading to exclusion of 174 individuals. Since sleep patterns are different in bipolar individuals with manic episodes (i.e., decreased need for sleep) compared to those with depressive episodes (i.e., increased need for sleep), we excluded bipolar individuals with a current hypomanic or manic episode (*n* = 10). Finally, we yielded a sample size of *N* = 657 (HC = 267, AD = 390; 483 female, 166 male, eight diverse; *M*_age_ = 34.87, *SD*_age_ = 13.86). In the AD group, 50 stated to be diagnosed with a single-episode depression, 280 with recurrent depression, 49 with bipolar disorder, 10 with dysthymia, and one with an unspecified AD. Participants were invited via an online link. The survey took 20–30 min. The study was part of a large-scaled ongoing project (“Health-protective and harmful moderating effects of the Dark Triad Traits in Individuals with Affective Disorders and Mentally Healthy Individuals”). A preregistration of the major project can be found at AsPredicted.^1^ All participants gave written informed consent prior to participation. This study was conducted in accordance with the Declaration of Helsinki and was approved by the Ethics Committee of the Medical University Graz (EC-number: 33-632 ex 20/21).

### 2.2. Measurements

This study was conducted via the survey tool LimeSurvey (version 3.28.39), which was opened from November 2021 to November 2022.

#### 2.2.1. Socio-demographic questionnaire

Participants were asked about personal information (e.g., gender), information on their AD (in the AD group), and medical information (e.g., comorbidities).

#### 2.2.2. Short dark triad

To assess the DT manifestation, we administered the Short Dark Triad (SD3; Jones and Paulhus, 2014), which comprises 27 items. Nine items for each trait were presented as statements (e.g., Machiavellianism: *“Most people can be manipulated.”*). Participants rated their agreement on a five-point Likert scale, ranging from (1) = “disagree strongly” to (5) = “agree strongly.” Scale scores were constructed by calculating the mean of the corresponding items (score range = 0–5, higher values indicate higher manifestations of the respective trait). All scales indicated sufficient internal consistency (Cronbach’s α for narcissism: α = 0.71, Machiavellianism: α = 0.79, psychopathy: α = 0.71; all α values were calculated for the results obtained in the current study).

#### 2.2.3. Pittsburgh sleep quality index

To assess sleep quality, we administered the Pittsburgh Sleep Quality Index (PSQI; Buysse et al., 1989), which assesses subjective sleep problems throughout the previous month. It comprises 19 items, which generate seven component scores (range 0–3, higher scores indicate worse sleep quality): sleep quality, sleep latency, sleep duration, habitual sleep efficiency, sleep disturbances, use of sleep medication, and daytime sleepiness. To construct a total score, the sum of the component scores was built (score range = 0–21). Cronbach’s α, which was calculated for the results obtained in the current study, indicated good internal consistency (α = 0.80).

### 2.3. Statistics

All analyses were conducted in IBM SPSS Statistics 28. For descriptive purposes, group differences in demographic and psychometric variables were calculated using *χ*^2^-tests and *t*-tests. Subsequently, moderation analyses assessed whether the relationships between group (HC vs. AD) and sleep quality were moderated by (1) narcissism, (2) Machiavellianism, and (3) psychopathy. Since a previous study on the DT and sleep quality included sex and age as covariates in a mediation analysis (Yang et al., 2019), we aimed to control for both variables. Thus, the relationships of age, gender, and the study variables were determined with preliminary Bonferroni-Holm corrected correlation analyses [adjusted initial α-level of 0.01 for *n* = 7 tests (0.05/7; Holm, 1979)]. We used the PROCESS Macro v4.1 (Hayes, 2022) to estimate the moderation models and applied 95% BCa bootstrapping confidence intervals based on 5,000 samples. Means were centered prior to analyses. Significant interaction effects were probed with simple slope analyses. To determine low, average, and high levels of the moderator variables, we used the 16th, 50th, and 84th variable percentiles. Hypotheses were tested two-tailed and statistical assumptions were met. Data and analysis scripts can be accessed via https://doi.org/10.17605/OSF.IO/2Y4A9.

## 3. Results

Descriptive statistics of are depicted in Table 1.

**Table 1:** Group differences in demographic and psychometric properties between individuals with affective disorders (AD) and healthy controls (HC).

Variable	Group		Total (*n* = 657)	Statistics	
	HC (*n* = 267)	AD (*n* = 390)		*χ* ^2^	*t*
Age	*M* = 29.82	*M* = 38.33	*M* = 34.87		**−9.10** ^***^
	*SD* = 10.01	*SD* = 15.00	*SD* = 13.86		
Gender				5.55	
Female	199 (74.53%)	284 (72.82%)	483 (73.51%)		
Male	68 (25.47%)	98 (25.13%)	166 (25.27%)		
Diverse	0 (0.0%)	8 (2.05%)	8 (1.22%)		
Education				**128.49** ^***^	
No formal education	0 (0%)	1 (0.26%)	1 (0.15%)		
Compulsory schooling	2 (0.75%)	43 (11.02%)	45 (6.84%)		
Apprenticeship	19 (7.12%)	112 (28.72%)	131 (19.94%)		
High school diploma	88 (32.96%)	148 (37.95%)	236 (35.92%)		
Bachelor degree	88 (32.96%)	34 (8.72%)	121 (18.42%)		
Master degree	60 (22.47%)	46 (11.79%)	106 (16.13%)		
PhD	10 (3.75%)	7 (1.79%)	17 (1.67%)		
Somatic comorbidities (yes)	19 (7.12%)	141 (36.15%)	160 (24.35%)	**72.54** ^***^	
Psychiatric comorbidities (yes)	0 (0.0%)	205 (52.56%)	205 (31.20%)	**204.00** ^***^	
Somatic medication (yes)	18 (6.74%)	146 (37.44%)	164 (24.96%)	**79.72** ^***^	
Psychiatric medication (yes)	0 (0.0%)	330 (84.62%)	330 (50.23%)	**453.92** ^***^	
FDR (yes)	0 (0.0%)	148 (37.95%)	148 (22.53%)	**130.78** ^***^	
Narcissism	*M* = 2.41	*M* = 2.12	*M* = 2.24		**5.62** ^***^
	*SD* = 0.62	*SD* = 0.68	*SD* = 0.67		
Machiavellianism	*M* = 2.42	*M* = 2.55	*M* = 2.50		**−2.15** ^*^
	*SD* = 0.68	*SD* = 0.81	*SD* = 0.76		
Psychopathy	*M* = 1.77	*M* = 1.89	*M* = 1.84		**−2.41** ^*^
	*SD* = 0.56	*SD* = 0.66	*SD* = 0.63		
Sleep quality	*M* = 4.61	*M* = 10.01	*M* = 7.81		**−18.27** ^***^
(PSQI Score)	*SD* = 2.51	*SD* = 4.36	*SD* = 4.57		

M, Mean; SD, Standard deviation; FDR, First-degree relative diagnosed with a psychiatric disorder; HC, Healthy controls; AD, Individuals with affective disorders; PSQI, Pittsburgh sleep quality index (higher scores indicate worse sleep quality). ^*^p < 0.05, ^***^p < 0.001. Significant results are printed in bold.

We performed Bonferroni-Holm corrected Pearson-and Spearman correlation analyses between the variables gender, age, and presence of AD, DT, and PSQI score (see Table 2). For narcissism, we observed that males and HC tended to demonstrate higher values. Regarding Machiavellianism, men scored significantly higher than women. Higher psychopathy values were found in individuals younger in age and males. Further, elder individuals and those with AD reported worse sleep quality.

**Table 2:** Pearson-and Spearman correlation analyses between demographic variables, Dark Triad traits, and sleep quality.

Variable	Age	Gender^a^	Group^a^
Narcissism	−0.05	**0.20** ^***^	**−0.22** ^***^
Machiavellianism	−0.02	**0.10** ^*^	0.06
Psychopathy	**−0.12** ^*^	**0.24** ^***^	0.08
PSQI Score	**0.26** ^***^	0.03	**0.61** ^***^

Gender = Female (=1), male (=2), and diverse (=3). Group, Healthy controls (= 1) vs. individuals with affective disorders (=2). PSQI, Pittsburgh sleep quality index (higher scores indicate worse sleep quality). *N* = 649. ^*^p < 0.05, ^***^p < 0.001. Bonferroni-Holm correction with initial α = 0.01 for all correlations. Significant results are printed in bold. ^a^Spearman correlation coefficients.

To investigate whether the relationship between group (AD vs. HC) and PSQI score is moderated by the DT, we conducted three moderation analyses with the outcome variable PSQI score, the predictor variable group (AD vs. HC), the moderating variables narcissism, Machiavellianism, and psychopathy, and the covariates age and gender. For narcissism, no significant interaction effect between group and PSQI score was found (*b* = −0.20, 95% CI [−1.02; 0.57], *t* = −0.50, *p* = 0.620). Regarding Machiavellianism and psychopathy, a significant negative interaction effect in the relationship between group and PSQI score was found, regardless of age and gender (Machiavellianism: *b* = −0.76, 95% CI [−1.45; −0.08], *t* = −2.12, *p* < 0.05; psychopathy: *b* = −1.15, 95% CI [−2.01; −0.26], *t* = −2.59, *p* < 0.05). This indicates that with increasing levels of Machiavellianism and psychopathy, the difference in sleep quality between individuals with AD and HC decreases. Moderation statistics are depicted in Table 3.

**Table 3:** Linear model of the predictors of sleep quality.

	*b*	*SE_b_*	*t*	*p*
Constant	**−1.55**	0.60	−2.60	0.010
	[−2.74; −0.40]			
Group	**5.15**	0.29	17.97	0.000
	[4.59; 5.71]			
Narcissism^a^	0.29	0.61	0.48	0.635
	[−0.90; 1.48]			
Group × Narcissism	−0.20	0.41	−0.50	0.620
	[−1.02; 0.57]			
Constant	**−1.27**	0.61	−2.09	0.037
	[−2.48; −0.10]			
Group	**5.08**	0.28	17.93	0.000
	[4.53; 5.63]			
Machiavellianism^a^	**1.66**	0.56	2.97	0.003
	[0.61; 2.72]			
Group × Machiavellianism	**−0.76**	0.36	−2.12	0.034
	[−1.45; −0.08]			
Constant	−1.15	0.61	−1.89	0.060
	[−2.34; 0.04]			
Group	**5.04**	0.29	17.68	0.000
	[4.49; 5.58]			
Psychopathy^a^	**2.39**	0.70	3.43	0.001
	[1.01; 3.74]			
Group × Psychopathy	**−1.15**	0.44	−2.58	0.010
	[−2.01; −0.26]			

Group, Healthy controls (=1) vs. individuals with affective disorders (=2). Linear Model controlling for age and gender. Total *R*^2^ for narcissism, Machiavellianism, and psychopathy = 0.35. 95% BCa bootstrapping confidence intervals based on 5,000 samples in square brackets. Significant results are printed in bold. ^a^Mean centered variables.

**Figure 1:**
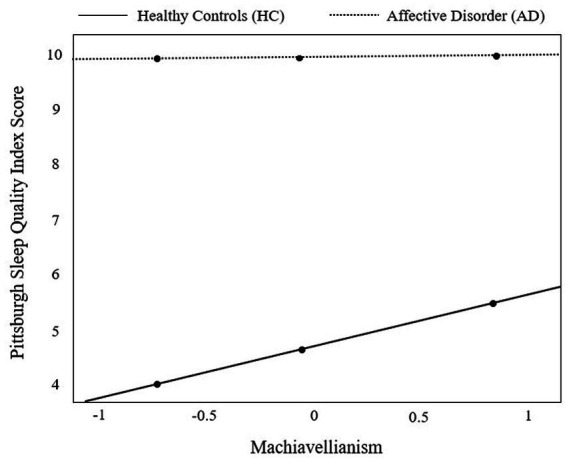
Visual representation of the moderation effect of group [individuals with affective disorders (AD) vs. healthy controls (HC)] on sleep quality (as measured by the Pittsburgh Sleep Quality Index) by Machiavellianism. The moderator variable Machiavellianism was centered prior to moderation analysis. Results from probing the moderator at the 16th, 50th, and 84th percentile (black dots) revealed a significant positive effect of group (HC vs. AD) on the Pittsburgh Sleep Quality Index at low [*θAD→PSQI*|(Machiavellianism = −0.72) = 5.63, CI [4.87; 6.38], *t* = 14.65, *p* < 0.001], average [*θAD→PSQI*|(Machiavellianism = −0.05) = 5.12, CI [4.46; 5.67], *t* = 18.02, *p* < 0.001], and high manifestations of Machiavellianism [*θAD→PSQI*|(Machiavellianism = 0.84) = 4.44, CI [3.63; 5.25], *t* = 10.77, *p* < 0.001].

To examine the significant interaction effects of *group × Machiavellianism* on sleep quality (PSQI), we conducted a simple slope analysis. Group had a significant positive effect on the PSQI score at low [*θ_AD → PSQI_*|(Machiavellianism = −0.72) = 5.63, CI [4.87; 6.38], *t* = 14.65, *p* < 0.001], average [*θ_AD → PSQI_*|(Machiavellianism = −0.05) = 5.12, CI [4.46; 5.67], *t* = 18.02, *p* < 0.001], and high manifestations of Machiavellianism [*θ_AD → PSQI_*|(Machiavellianism = 0.84) = 4.44, CI [3.63; 5.25], *t* = 10.77, *p* < 0.001]. At each examined level of Machiavellianism, sleep quality was worse in individuals with AD. Figure 1 reveals that Machiavellianism seems to play a more pronounced role in sleep quality deterioration in HC than in AD.

**Figure 2:**
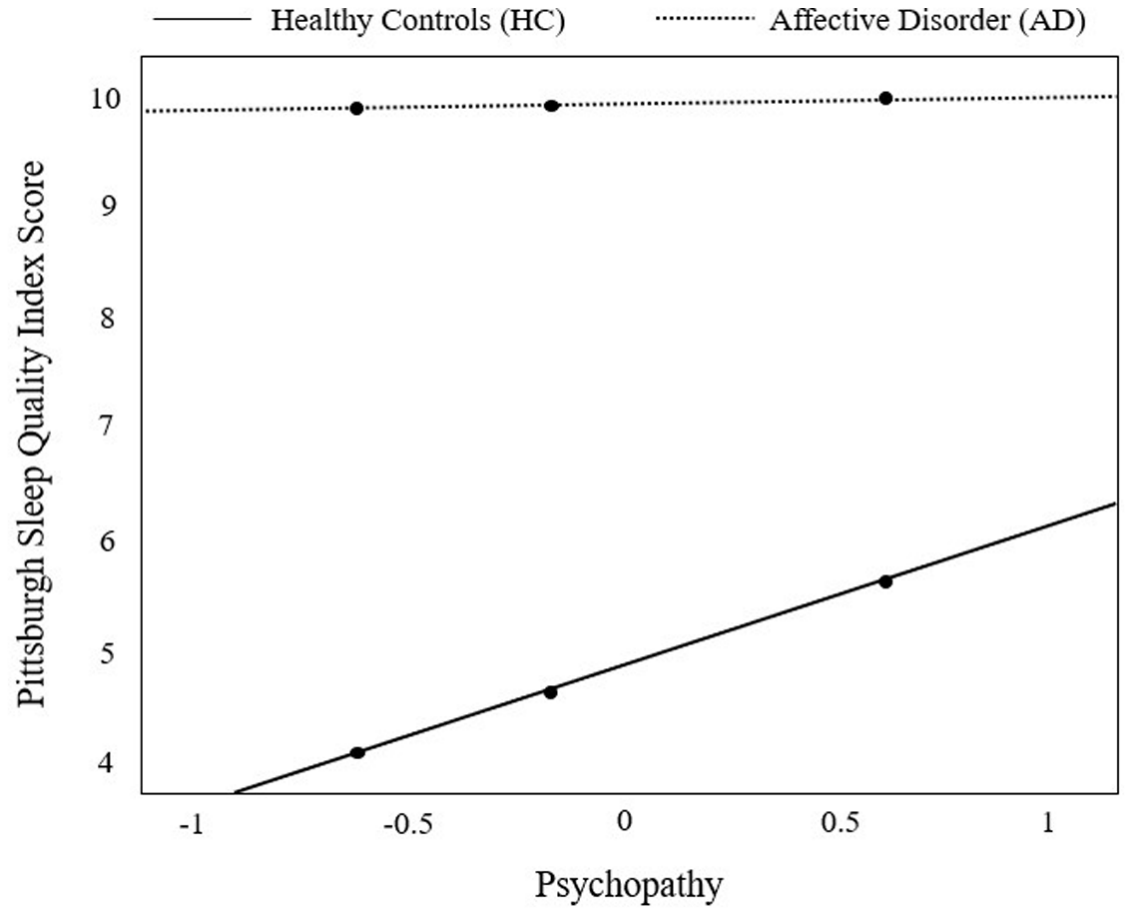
Visual representation of the moderation effect of group [individuals with affective disorders (AD) vs. healthy controls (HC)] on sleep quality (as measured by the Pittsburgh Sleep Quality Index) by psychopathy. The moderator variable psychopathy was centered prior to moderation analysis. Results from probing the moderator at the 16th, 50th, and 84th percentile (black dots) revealed a significant positive effect of group (HC vs. AD) on the Pittsburgh Sleep Quality Index [*θAD→PSQI*|(psychopathy = −0.62) = 5.75, CI [4.96; 6.54], *t* = 14.34, *p* < 0.001], average [*θAD→PSQI*|(psychopathy = −0.17) = 5.24, CI [4.66; 5.83], *t* = 17.62, *p* < 0.001], and high manifestations of psychopathy [*θAD→PSQI*|(psychopathy = 0.60) = 4.35, CI [3.59; 5.11], *t* = 11.28, *p* < 0.001].

Regarding the interaction effect of *group* × *psychopathy* on sleep quality (PSQI score), the simple slope analysis demonstrated a significant positive effect of group on the PSQI score at low [*θ_AD → PSQI_*|(psychopathy = −0.62) = 5.75, CI [4.96; 6.54], *t* = 14.34, *p* < 0.001], average [*θ_AD → PSQI_*|(psychopathy = −0.17) = 5.24, CI [4.66; 5.83], *t* = 17.62, *p* < 0.001], and high manifestations of psychopathy [*θ_AD → PSQI_*|(psychopathy = 0.60) = 4.35, CI [3.59; 5.11], *t* = 11.28, *p* < 0.001]. Sleep quality was worse in the AD group than in the HC group at each examined level of psychopathy. Figure 2 visualizes that psychopathy seems to play a more pronounced role in sleep quality deterioration in HC than in AD.

## 4. Discussion

This study investigated the moderating effect of narcissism, Machiavellianism, and psychopathy in the relationship between AD (as opposed to HC) and sleep quality. Results reveal that Machiavellianism and psychopathy, but not narcissism, are differently associated to sleep quality in individuals with AD and HC. In HC, there is a more profound deterioration in sleep quality, depending on the individual manifestation of Machiavellianism and psychopathy, compared to AD.

Our hypothesis of narcissism posing as a significant negative moderator in the relationship between group and sleep quality was not supported by our results, since narcissism did not have a significant influence on this relationship in our study. However, this corroborates previous research on the DT and sleep behavior, which indicates no association between narcissism and sleep–wake habits (Rahafar et al., 2022), chronotype (Jonason et al., 2013), sleep disturbances (Sabouri et al., 2016), and insomnia (Akram et al., 2018; Yang et al., 2019). One explanation for this finding is that the negative relationship between the DT and sleep disturbances exists due to negatively affected cognitive-emotional processes (e.g., rumination; Sabouri et al., 2016), which is also present in AD (e.g., Kovács et al., 2020). However, individuals high in narcissism are less inclined to negative rumination due to their subjective grandiosity (Yang et al., 2019). Of all the DT, narcissism has even been determined as a buffer for negative health outcomes (Jonason et al., 2015a), but might be more relevant in other health-related behaviors. Another explanation could be the lacking differentiation between narcissism subfacets in our study. For example, Jonason et al. (2013) found a positive association between an eveningness disposition and exploitative narcissism. Similarly, vulnerable narcissism was associated with poor sleep quality (Annen et al., 2017). Differentiating between narcissism subfacets could elucidate the relationship of narcissism and sleep quality.

Further, we hypothesized that both Machiavellianism and psychopathy significantly negatively moderate the relationship between group and sleep quality, which was corroborated by our results. Indeed, Machiavellianism and psychopathy were found to pose as negative moderating variables in this relationship, indicating that these traits have a negative influence on sleep quality in both AD and HC. This is consistent with previous literature, which demonstrates the deteriorating effect of the so-called “Malicious Two” traits on insomnia symptoms (Akram et al., 2018; Yang et al., 2019), sleep disturbances (Sabouri et al., 2016), sleep and wake-up time instability (Rahafar et al., 2022), and chronotypes (Jonason et al., 2013; Costa Porfírio and Corrêa Varella, 2022). Deteriorated sleep among those high in psychopathy and Machiavellianism may be a result of insufficient emotion regulation, resulting in negatively toned cognitive activity, which affects sleep quality (Sabouri et al., 2016; Akram et al., 2018). Further, Machiavellianism and psychopathy were previously connected to cortisol levels (Dane et al., 2018), and psychopathy was associated with altered cortisol awakening responses, which could affect sleep quality (Johnson et al., 2014). These findings suggest an underlying biological pathway and highlight the interaction between personality traits, physiological aspects, and behavior. At higher levels of Machiavellianism and psychopathy, there was a more profound association between these traits and deteriorated sleep quality in HC compared to AD. Moreover, the sleep quality score only slightly changed across the personality trait manifestations in AD. This indicates that the contribution of these traits to sleep quality may be insignificant in the AD group, given the generally poorer sleep quality in AD compared to HC. Possibly, there are other variables playing a more important role in the sleep quality of individuals with AD than these traits, but do not affect HC. For instance, HC are not influenced by illness-related variables (e.g., symptomatology, Gruber et al., 2011; Becker Cretu et al., 2016), or psychopharmacological medication (Barton et al., 2022), which might affect sleep quality in AD. Moreover, worse sleep quality was previously associated with deteriorated neurocognitive functioning (Russo et al., 2015), less social relationships (Kent et al., 2015), poor health behaviors (Lee et al., 2020), and more stressful life-events (Saunders et al., 2013). All of these factors are more present in individuals with AD compared to HC, thus possibly determining sleep quality in AD rather than personality traits. Future studies should look into the exact determinants of deteriorated sleep quality in both AD and HC.

Since the previously mentioned studies were conducted in samples of HC, the current study is the first to provide data on the negative influence of Machiavellianism and psychopathy on sleep quality in individuals with AD and HC. Considering these traits could result in a more personalized treatment of sleep disorders, which could lead to an amelioration of AD. Nevertheless, Machiavellianism and psychopathy also seem to profoundly influence sleep quality of mentally healthy individuals. Thus, the individual manifestation of these traits could serve the early detection and prevention of sleep deterioration in both groups.

### 4.1. Limitations

The study results should be interpreted with following limitations in mind. First, our statistical approach does only allow for an interpretation of the DT manifestation in sleep quality in both groups (AD vs. HC). Hence, a between-group difference cannot be statistically determined. Given the cross-sectional design of the current study, the results lack causality and direction. Moreover, the use of self-assessment questionnaires implies the possibility of motivation and social desirability biases, and the use of self-reported diagnoses might result in incorrect or inaccurate participant information. The study investigated the Dark Triad traits as it is one of the most examined personality constructs, however, future studies might investigate the Dark Tetrad (including the Sadism trait; Paulhus, 2014), as it has also been shown to be negatively associated with various health domains (e.g., Kircaburun et al., 2018; Konc et al., 2022). Moreover, future research should look into the diagnosis of AD as a moderator between the DT and sleep quality, since the DT serve as risk factors for both AD and poor sleep quality. Finally, the study was conducted online, thus the participation setting might not have been entirely equal for all participants, and some subpopulations might have had a more facilitated access than others, hence limiting generalizability of the outcomes.

### 4.2. Conclusion

This study is the first to examine the DT with regards to sleep quality in a sample of individuals with AD and HC. It was found that Machiavellianism and psychopathy, but not narcissism, negatively affect sleep quality in both groups. This seems to be more pronounced in mentally healthy individuals. Our results emphasize the role of personality traits in the multifactorial aetiopathogenesis of AD and point out their consideration in the treatment or prevention of AD symptomatology (e.g., sleep disturbances). Incorporating personality traits could support the creation of customized treatment plans, thereby reducing treatment duration, strengthening adherence, and optimizing treatment outcomes.

## Data availability statement

The datasets presented in this study can be found in online repositories. The names of the repository/repositories and accession number(s) can be found at: Open Science Frame repository (OSF.io; https://doi.org/10.17605/OSF.IO/2Y4A9).

## Ethics statement

The studies involving humans were approved by Ethics Committee of the Medical University Graz. The studies were conducted in accordance with the local legislation and institutional requirements. The participants provided their written informed consent to participate in this study.

## Author contributions

ES: conceptualization, methodology, software, validation, formal analysis, investigation, data curation, writing-original draft, writing—review and editing, visualization, and project administration. ND: conceptualization, methodology, validation, investigation, resources, writing—review and editing, supervision, and project administration. DL: conceptualization, writing—review and editing, and supervision. ER: conceptualization, resources, writing—review and editing, supervision, and project administration. AB: conceptualization, methodology, validation, formal analysis, investigation, resources, writing—review and editing, supervision, and project administration. All authors contributed to the article and approved the submitted version.

## Funding

The work was supported by the Open Access publication cost funding program of the Province of Styria, Austria (ABT12-174904/2023).

## Conflict of interest

The authors declare that the research was conducted in the absence of any commercial or financial relationships that could be construed as a potential conflict of interest.

## Publisher’s note

All claims expressed in this article are solely those of the authors and do not necessarily represent those of their affiliated organizations, or those of the publisher, the editors and the reviewers. Any product that may be evaluated in this article, or claim that may be made by its manufacturer, is not guaranteed or endorsed by the publisher.

## Footnotes

^1^ aspredicted.org/blind.php?x=6PD_1LZ

## References

[ref1] Akram U. Allen S. McCarty K. Gardani M. Tan A. Villarreal D.. (2018). The relationship between insomnia symptoms and the dark triad personality traits. J. Pers. Individ. Differ. 131, 212–215. doi: 10.1016/j.paid.2018.05.001

[ref2] Annen H. Nakkas C. Bahmani D. S. Gerber M. Brand S. (2017). Vulnerable narcissism as key link between dark triad traits, mental toughness, sleep quality and stress. Eur. Psychiatry 41:S261. doi: 10.1016/j.eurpsy.2017.02.070

[ref3] Bader M. Hilbig B. E. Zettler I. Moshage M. (2022). Rethinking aversive personality: decomposing the dark triad traits into their common core and unique flavors. J. Pers. 91, 1–26. doi: 10.1111/jopy.12785 36256568

[ref4] Barton J. Mio M. Timmins V. Mitchell R. H. B. Murray B. J. Goldstein B. I. (2022). Factors associated with sleep disturbance amongst youth with bipolar disorder. J. Can. Acad. Child Adolesc. Psychiatry 31, 165–175. PMID: 36425019 PMC9661909

[ref5] Becker Cretu J. Culver J. L. Goffin K. C. Shah S. Ketter T. A. (2016). Sleep, residual mood symptoms, and time to relapse in recovered patients with bipolar disorder. J. Affect. Disord. 190, 162–166. doi: 10.1016/j.jad.2015.09.076 26519636

[ref6] Bianchi R. Mirkovic D. (2020). Is Machiavellianism associated with depression? A cluster-analytic study. J. Pers. Individ. Differ. 152:109594. doi: 10.1016/j.paid.2019.109594

[ref7] Book A. Visser B. A. Volk A. A. (2015). Unpacking "evil": claiming the core of the dark triad. J. Pers. Individ. Differ. 73, 29–38. doi: 10.1016/j.paid.2014.09.016

[ref8] Buysse D. J. Reynolds C. F. Monk T. H. Berman S. R. Kupfer D. J. (1989). The Pittsburgh sleep quality index: a new instrument for psychiatric practice and research. Psychiatry Res. 28, 193–213. doi: 10.1016/0165-1781(89)90047-4 2748771

[ref9] Costa Porfírio J. Corrêa Varella M. (2022). Testing the cognitive niche hypothesis with structural equation modeling: different dark traits predict an evening-chronotype in males and females. Curr. Psychol. doi: 10.1007/s12144-022-04111-w

[ref10] Dalkner N. Bengesser S. A. Birner A. Fellendorf F. T. Fleischmann E. Großschädl K.. (2021). Metabolic syndrome impairs executive function in bipolar disorder. Front. Neurosci. 15:717824. doi: 10.3389/fnins.2021.717824, PMID: 34456679 PMC8385126

[ref11] Dalkner N. Reininghaus E. Z. Riedrich K. Rieger A. Birner A. Fellendorf F. T.. (2018). Psychopathic personality factor “fearless dominance” is related to low self-reported stress-levels, fewer psychiatric symptoms, and more adaptive stress coping in psychiatric disorders. Psychiatry Res. 270, 68–77. doi: 10.1016/j.psychres.2018.09.018, PMID: 30245379

[ref12] Dallaspezia S. Benedetti F. (2015). Sleep deprivation therapy for depression. Curr. Top. Behav. Neurosci. 25, 483–502. doi: 10.1007/7854_2014_363 25549913

[ref13] Dane L. K. Jonason P. K. McCaffrey M. (2018). Physiological tests of the cheater hypothesis for the dark triad traits: testosterone, cortisol, and a social stressor. J. Pers. Individ. Differ. 121, 227–231. doi: 10.1016/j.paid.2017.09.010

[ref14] Dębska M. Dębski P. Polechoński J. Rozpara M. Tomik R. (2021). The dark triad of personality in the context of health behaviors: ally or enemy? Int. J. Environ. Res. Public Health 18:4113. doi: 10.3390/ijerph18084113, PMID: 33924649 PMC8069531

[ref15] Ellison W. D. Levy K. N. Cain N. M. Ansell E. B. Pincus A. L. (2013). The impact of pathological narcissism on psychotherapy utilization, initial symptom severity, and early-treatment symptom change: a naturalistic investigation. J. Pers. Assess. 95, 291–300. doi: 10.1080/00223891.2012.742904 23186259

[ref16] Fang H. Tu S. Sheng J. Shao A. (2019). Depression in sleep disturbance: a review on a bidirectional relationship, mechanisms and treatment. J. Cell. Mol. Med. 23, 2324–2332. doi: 10.1111/jcmm.14170, PMID: 30734486 PMC6433686

[ref17] Faul F. Erdfelder E. Lang A.-G. Buchner A. (2007). G*power 3: a flexible statistical power analysis program for the social, behavioral, and biomedical sciences. Behav. Res. Methods 39, 175–191. doi: 10.3758/bf03193146, PMID: 17695343

[ref18] Gold A. K. Sylvia L. G. (2016). The role of sleep in bipolar disorder. Nat. Sci. Sleep 8, 207–214. doi: 10.2147/NSS.S85754, PMID: 27418862 PMC4935164

[ref19] Gómez-Leal R. Megías-Robles A. Gutiérrez-Cobo M. J. Cabello R. Fernández-Abascal E. G. Fernández-Berrocal P. (2019). Relationship between the dark triad and depressive symptoms. PeerJ. 7, e8120–e8127. doi: 10.7717/peerj.8120, PMID: 31803535 PMC6886484

[ref20] Gruber J. Miklowitz D. J. Harvey A. G. Frank E. Kupfer D. Thase M. E.. (2011). Sleep matters: sleep functioning and course of illness in bipolar disorder. J. Affect. Disord. 134, 416–420. doi: 10.1016/j.jad.2011.05.016, PMID: 21683450 PMC3387668

[ref21] Hayes A. F. (2022). PROCESS macro for SPSS, SAS, and R. Available at: https://www.processmacro.org/index.html (Accessed June 15, 2023).

[ref22] Holm S. (1979). A simple sequentially Rejective multiple test procedure. Scand. J. Stat. 6, 65–70.

[ref23] Huang V. Peck K. Mallya S. Lupien S. J. Fiocco A. J. (2016). Subjective sleep quality as a possible mediator in the relationship between personality traits and depressive symptoms in middle-aged adults. PLoS One 11, 1–18. doi: 10.1371/journal.pone.0157238, PMID: 27285159 PMC4902234

[ref24] Irandoust K. Taheri M. (2018). Effects of different daytime exercises on the quality of sleep and appetite of obese women. Int. Arch. Health Sci. 5, 111–114. doi: 10.4103/iahs.iahs_41_18

[ref25] Johnson M. M. Caron K. M. Mikolajewski A. J. Shirtcliff E. A. Eckel L. A. Taylor J. (2014). Psychopathic traits, empathy, and aggression are differentially related to cortisol awakening response. J. Psychopathol. Behav. Assess. 36, 380–388. doi: 10.1007/s10862-014-9412-7

[ref26] Jonason P. K. Baughman H. M. Carter G. L. Parker P. (2015a). Dorian gray without his portrait: psychological, social, and physical health costs associated with the dark triad. J. Pers. Individ. Differ. 78, 5–13. doi: 10.1016/j.paid.2015.01.008

[ref27] Jonason P. K. Duineveld J. J. Middleton J. P. (2015b). Pathology, pseudopathology, and the dark triad of personality. J. Pers. Individ. Differ. 78, 43–47. doi: 10.1016/j.paid.2015.01.028

[ref28] Jonason P. K. Jones A. Lyons M. (2013). Creatures of the night: Chronotypes and the dark triad traits. J. Pers. Individ. Differ. 55, 538–541. doi: 10.1016/j.paid.2013.05.001

[ref29] Jones D. N. Paulhus D. L. (2014). Introducing the short dark triad (SD3): a brief measure of dark personality traits. Assessment 21, 28–41. doi: 10.1177/1073191113514105 24322012

[ref30] Kent R. G. Uchino B. N. Cribbet M. R. Bowen K. Smith T. W. (2015). Social relationships and sleep quality. Ann. Behav. Med. 49, 912–917. doi: 10.1007/s12160-015-9711-6, PMID: 25976874 PMC4636437

[ref31] Khcharem A. Souissi M. Sahnoun Z. (2022). Effects of repeated low-dose caffeine ingestion during a night of total sleep deprivation on endurance performance and psychological state in young recreational runners. Int. J. Sport Stud. Health 4:e123038. doi: 10.5812/intjssh.123038 35791877

[ref32] Kircaburun K. Jonason P. K. Griffiths M. D. (2018). The dark tetrad traits and problematic online gaming: the mediating role of online gaming motives and moderating role of game types. J. Pers. Individ. Differ. 135, 298–303. doi: 10.1016/j.paid.2018.07.038

[ref33] Kline C. (2013). “Sleep quality” in Encyclopedia of Behavioral Medicine. eds. Gellman M. D. Turner J. R. (New York, NY: Springer), 1811–1813.

[ref34] Konc I. Petrović K. Dinić B. M. (2022). Dark tetrad and COVID-19 protective measures: mediating effects of risk-taking tendencies. J. Pers. Individ. Differ. 186:111341. doi: 10.1016/j.paid.2021.111341, PMID: 34744234 PMC8563827

[ref35] Kovács L. N. Takacs Z. K. Tóth Z. Simon E. Schmelowszky Á. Kökönyei G. (2020). Rumination in major depressive and bipolar disorder – a meta-analysis. J. Affect. Disord. 276, 1131–1141. doi: 10.1016/j.jad.2020.07.131, PMID: 32777651

[ref36] Lee S. Y. Ju Y. J. Lee J. E. Kim Y. T. Jong S. C. Choi Y. J.. (2020). Factors associated with poor sleep quality in the Korean general population: providing information from the Korean version of the Pittsburgh sleep quality index. J. Affect. Disord. 271, 49–58. doi: 10.1016/j.jad.2020.03.069, PMID: 32312697

[ref37] Lopresti A. L. Hood S. D. Drummond P. D. (2013). A review of lifestyle factors that contribute to important pathways associated with major depression: diet, sleep and exercise. J. Affect. Disord. 148, 12–27. doi: 10.1016/j.jad.2013.01.014, PMID: 23415826

[ref38] Malesza M. Kaczmarek M. C. (2019). Dark side of health-predicting health behaviors and diseases with the dark triad traits. J. Public Health 29, 275–284. doi: 10.1007/s10389-019-01129-6

[ref39] Ng T. H. Chung K. F. Ho F. Y. Y. Yeung W. F. Yung K. P. Lam T. H. (2015). Sleep-wake disturbance in interepisode bipolar disorder and high-risk individuals: a systematic review and meta-analysis. Sleep Med. Rev. 20, 46–58. doi: 10.1016/j.smrv.2014.06.006, PMID: 25060968

[ref40] Nutt D. J. Wilson S. Paterson L. (2008). Sleep disorders as core symptoms of depression. Dialogues Clin. Neurosci. 10, 329–336. doi: 10.31887/dcns.2008.10.3/dnutt, PMID: 18979946 PMC3181883

[ref41] Palagini L. Cipollone G. Masci I. Caruso D. Paolilli F. Perugi G.. (2019). Insomnia symptoms predict emotional dysregulation, impulsivity and suicidality in depressive bipolar II patients with mixed features. Compr. Psychiatry 89, 46–51. doi: 10.1016/j.comppsych.2018.12.009, PMID: 30593973

[ref42] Paulhus D. L. (2014). Toward a taxonomy of dark personalities. Curr. Dir. Psychol. Sci. 23, 421–426. doi: 10.1177/0963721414547737

[ref43] Paulhus D. L. Williams K. M. (2002). The dark triad of personality: narcissism, Machiavellianism, and psychopathy. J. Res. Pers. 36, 556–563. doi: 10.1016/S0092-6566(02)00505-6

[ref44] Platzer M. Fellendorf F. T. Bengesser S. A. Birner A. Dalkner N. Hamm C.. (2021). The relationship between food craving, appetite-related hormones and clinical parameters in bipolar disorder. Nutrients 13:76. doi: 10.3390/nu13010076 PMC7824587 33383670

[ref45] Rahafar A. Kalbacher L. S. Randler C. (2022). A closer look at the sleep/wake habits and dark triad traits. Appl. Sci. 12:5963. doi: 10.3390/app12125963

[ref46] Rahimi A. Ahmadpanah M. Shamsaei F. Cheraghi F. Sadeghi Bahmani D. Holsboer-Trachsler E.. (2016). Effect of adjuvant sleep hygiene psychoeducation and lorazepam on depression and sleep quality in patients with major depressive disorders: results from a randomized three-arm intervention. Neuropsychiatr. Dis. Treat. 12, 1507–1515. doi: 10.2147/NDT.S110978, PMID: 27382293 PMC4922769

[ref47] Russo M. Mahon K. Shanahan M. Ramjas E. Solon C. Purcell S. M.. (2015). The relationship between sleep quality and neurocognition in bipolar disorder. J. Affect. Disord. 187, 156–162. doi: 10.1016/j.jad.2015.08.009, PMID: 26339925 PMC4598049

[ref48] Sabouri S. Gerber M. Lemola S. Becker S. P. Shamsi M. Shakouri Z.. (2016). Examining dark triad traits in relation to sleep disturbances, anxiety sensitivity and intolerance of uncertainty in young adults. Compr. Psychiatry 68, 103–110. doi: 10.1016/j.comppsych.2016.03.012, PMID: 27234190

[ref49] Saunders E. Novick D. M. Fernandez-Mendoza J. Kamali M. Ryan K. A. Langenecker S. A.. (2013). Sleep quality during euthymia in bipolar disorder: the role of clinical features, personality traits, and stressful life events. Int. J. Bipolar Disord. 1, 1–12. doi: 10.1186/2194-7511-1-16, PMID: 25505683 PMC4230686

[ref50] Shen K. (2022). The dark triad and depressive symptoms among chinese adolescents: moderated mediation models of age and emotion regulation strategies. Curr. Psychol., 1–10. doi: 10.1007/s12144-022-04132-5, PMID: 36531190 PMC9748877

[ref51] Shih S. I. Chi N. W. Wu C. C. Wang S. Y. (2021). When dark meets blue: the relations between dark triad personality and depression symptoms. Curr. Psychol. 40, 6110–6117. doi: 10.1007/s12144-019-00549-7

[ref52] Taheri M. Irandoust K. (2019). Morning exercise improves cognitive performance decrements induced by partial sleep deprivation in elite athletes. Biol. Rhythm. Res. 51, 644–653. doi: 10.1080/09291016.2019.1576279

[ref53] Walsh J. M. Atkinson L. A. Corlett S. A. Gurprit S. L. (2014). An insight into light as a chronobiological therapy in affective disorders. Chron. Physiol. Ther. 4, 79–85. doi: 10.2147/CPT.S56589

[ref54] Wittchen H.-U. Zaudig M. Fydrich T. (1997). Strukturiertes Klinisches Interview für DSM-IV [Structured Clinical Interview for DSM-IV]. Göttingen: Hogrefe

[ref55] World Health Organization (2022). Mental Disorders. Available at: https://www.who.int/news-room/fact-sheets/detail/mental-disorders (Accessed March 15, 2023).

[ref56] Yang M. Zhu X. Sai X. Zhao F. Wu H. Geng Y. (2019). The dark triad and sleep quality: mediating role of anger rumination. J. Pers. Individ. Differ. 151:109484. doi: 10.1016/j.paid.2019.06.027

